# Chronic Stability of Local Field Potentials Using Amorphous Silicon Carbide Microelectrode Arrays Implanted in the Rat Motor Cortex

**DOI:** 10.3390/mi14030680

**Published:** 2023-03-19

**Authors:** Eleanor N. Jeakle, Justin R. Abbott, Joshua O. Usoro, Yupeng Wu, Pegah Haghighi, Rahul Radhakrishna, Brandon S. Sturgill, Shido Nakajima, Teresa T. D. Thai, Joseph J. Pancrazio, Stuart F. Cogan, Ana G. Hernandez-Reynoso

**Affiliations:** 1 Department of Bioengineering, The University of Texas at Dallas, 800 W. Campbell Road, Richardson, TX 75080-3021, USA; 2 Department of Materials Science and Engineering, The University of Texas at Dallas, 800 W. Campbell Road, Richardson, TX 75080-3021, USA

**Keywords:** amorphous silicon carbide, microelectrode arrays, motor cortex, local field potentials

## Abstract

Implantable microelectrode arrays (MEAs) enable the recording of electrical activity of cortical neurons, allowing the development of brain-machine interfaces. However, MEAs show reduced recording capabilities under chronic conditions, prompting the development of novel MEAs that can improve long-term performance. Conventional planar, silicon-based devices and ultra-thin amorphous silicon carbide (a-SiC) MEAs were implanted in the motor cortex of female Sprague–Dawley rats, and weekly anesthetized recordings were made for 16 weeks after implantation. The spectral density and bandpower between 1 and 500 Hz of recordings were compared over the implantation period for both device types. Initially, the bandpower of the a-SiC devices and standard MEAs was comparable. However, the standard MEAs showed a consistent decline in both bandpower and power spectral density throughout the 16 weeks post-implantation, whereas the a-SiC MEAs showed substantially more stable performance. These differences in bandpower and spectral density between standard and a-SiC MEAs were statistically significant from week 6 post-implantation until the end of the study at 16 weeks. These results support the use of ultra-thin a-SiC MEAs to develop chronic, reliable brain-machine interfaces.

## 1. Introduction

Intracortical microelectrode arrays (MEAs) can record the electrical activity of neurons within the cortex, allowing the development of brain-machine interfacing devices [1,2,3,4,5]. However, the functionality of implantable brain-machine interfaces over time faces a significant challenge: the foreign body response in the brain that contributes to failure of the interface. This foreign body response is characterized by the migration of astrocytes, microglia, and macrophages seen as early as minutes following implantation [6,7,8]. As soon as 2 weeks post-implantation, this response has contributed to a significant decline in the detection of units during single unit analysis [3,7,9,10,11]. Local field potentials (LFPs) have been demonstrated as a robust alternative to single-unit analysis for the control of brain-machine interfaces [3,9,10,12,13]. LFPs are obtained by analyzing the lower frequencies of continuous electrophysiological recordings, generally using a low-pass filter below 300–500 Hz [10,14,15,16,17], compared to the frequency band for analyzing single units, which typically spans 300–10,000 Hz [10]. Rather than representing the firing of individual neurons, LFPs represent the overall synaptic activity at the recording site, as well as volume-conducted signals from more distant regions, and may be more stable overtime for analysis than single units [3,9,10,12,13,18,19,20]. LFPs have been used successfully in decoding applications both on their own [21,22] and in conjunction with single-unit analysis [23]. LFP-based decoding of cognitive state and motor intention can show accuracy on par with spike-based decoding [13,17,24]. Furthermore, recent studies have proposed the simultaneous use of LFPs and single units to improve chronic neural decoding performance [25].

Because of this, a major research goal is to design long-lasting MEAs that show reliable chronic LFP recordings [7,26,27]. Large cross-sectional areas of MEAs (>630 µm^2^) can lead to significant foreign body response and loss of neural recording [28,29,30]. A promising engineering approach to achieve chronic reliability of neural signals has been to reduce the cross-sectional area of implantable MEAs to largely evade the foreign body response [28,29,30]. A second approach to improve MEA reliability has been the use of materials such as amorphous silicon carbide (a-SiC). This is a corrosion-resistant, biocompatible material [31,32] that has been used as an encapsulation layer for the fabrication of MEAs [33,34,35]. Chronic implantation of a-SiC MEAs has been shown to not impact neuronal density, except in the immediate vicinity of the MEA, where the trauma of insertion may cause individual neurons to be displaced or mechanically damaged [32]. Recent studies have shown that a-SiC coating of conventional planar, silicon-based devices can yield significant improvements, including long-term neuronal survivability and high signal-to-noise ratio [36,37]. Furthermore, a-SiC is known to be stable as a dielectric material in a wet environment and resistant to dissolution [32], further supporting its use for the development of intracortical MEAs. In this study, we report on the use of ultra-thin a-SiC MEAs with a cross-sectional area of 160 µm^2^ to reduce foreign body response and improve chronic reliability of LFPs [38]. Ultra-thin a-SiC MEAs have been shown to successfully record single unit neural activity from the motor cortex in small and large animal models acutely (≤ 5 weeks post implantation) [11,35,39]. Here, we compare LFP recording performance of these devices with conventional MEAs for up to 16 weeks post-implantation. Our results indicate that ultra-thin a-SiC MEAs present a significant improvement to the existing conventional planar MEAs in the recording of LFPs beginning at 6 weeks post-implantation.

## 2. Materials and Methods

### 2.1. Devices

Experiments were performed using custom-built, a-SiC devices and compared to conventional planar multi-shank silicon devices. The a-SiC MEAs were fabricated using cleanroom microfabrication techniques as previously described [40] with four colinear shanks and four channels per shank. In brief, a base layer a-SiC was deposited on top of a sacrificial polyimide layer. A Ti/Au/Ti layer was deposited to serve as the underlying metal trace which interconnects between the Omnetics connector and electrode sites. A top layer of a-SiC was deposited. Then, small vias were etched in the top a-SiC layer to access the metal traces. Sputtered iridium oxide film (SIROF) was deposited at the electrode sites as a low impedance, high charge storage and injection capacity coating.

Each of the shanks was 2 mm long and designed to target the L4/L5 layers of the rat motor cortex. The microelectrode sites were spaced 200 µm beginning at 100 µm from the tip, positioned at 0.7, 0.5, 0.3, and 0.1 mm from the tip of each shank (i.e., all four electrode sites in each shank were localized within 700 µm from the tip; spanning 600 µm) with a 200 µm pitch between shanks. The penetrating shanks of the a-SiC devices were 8 µm thick by 20 µm wide, for a shank cross-sectional area of 160 µm^2^. These devices have been shown to penetrate rat pia mater without the necessity of a structural support or guide [41]. The MEAs were connected by conductive silver epoxy to a 16 channel Omnetics connector through a bond pad region located at the proximal end of the array, which was then encapsulated in medical-grade epoxy (EA M-121HP, Henkel Loctite, Germany). Completed devices were rejuvenated in phosphate buffered saline (PBS; Sigma-Aldrich, Saint Louis, MO, USA) by potential cycling to restore diminished electrochemical properties of the SIROF lost by the final fabrication steps.

Silicon-based conventional devices (A4x4-2mm-200-200-200-CM16LP, NeuroNexus Technologies, Ann Arbor, MI, USA) were custom-built to have a comparable length (2 mm), shank pitch (200 µm) and electrode site spacing (200 µm) to a-SiC MEAs. However, the cross-sectional area was larger at 630 µm^2^ (42 µm wide and 15 µm thick). The electrode sites of the conventional devices were fabricated with iridium metal and then activated prior to implantation to obtain activated iridium oxide film (AIROF) by a previously established process using a repeated pulsing protocol with alternating positive and negative bias [42].

### 2.2. Surgical Implantation of Neural Devices

All procedures, handling, and housing were approved by The University of Texas at Dallas Institutional Animal Care and Use Committee. Sixteen female Sprague–Dawley rats (Charles River Laboratories, Wilmington, MA, USA) were randomly assigned to two groups: (1) implanted with a-SiC MEAs (*n* = 5) and (2) silicon-based, conventional MEAs (*n* = 9). Implantation was performed following established procedures [43]. Briefly, animals were anesthetized using an intraperitoneal injection of ketamine (65 mg/kg), xylazine (13.33 mg/kg), and acepromazine (1.5 mg/kg) cocktail, followed by an intramuscular injection of atropine sulfate (0.093 mL/kg) (Covetrus, Portland, ME, USA). Ophthalmic ointment was applied to the eyes. The scalp was shaved to expose the skin. Anesthesia was confirmed by toe-pinch and maintained using isoflurane (0.5–1.5%) supplemented with pure O_2_ (500 mL/min). Anesthetized animals were fixed in a stereotactic frame. The animal’s temperature, respiration, and pulse were monitored throughout surgery. The animal’s scalp was sterilized with alternating 10% povidone-iodine solution and 70% alcohol followed by administration of 0.16 mL of 2% lidocaine (Covetrus Inc., Portland, ME, USA). Following an incision made to expose the skull and tissue resection, 3 anchoring bone screws were placed in the quadrants adjacent to the implant site. Quadrants were defined based on the bregma and suture line. A 2 mm by 2 mm craniotomy was made over the left motor cortex 2 mm anterior from the bregma and lateral to the suture line. The dura mater was resected to allow for implantation. Stainless steel ground and reference wires from the device were wrapped around the anchoring screws. MEAs were implanted using an electronically controlled micro-positioner (NeuralGlider, Actuated Medical, Inc., Ann Arbor, MI, USA) at 100 µm per second with ultrasonic actuation to a relative depth of 1.5 mm from the cortical surface so that all electrode sites were located only in layers L4 and L5 of the primary motor cortex (approximately 75% of electrode sites located within a single layer). A silicone elastomer (Kwik-Sil, World Precision Instruments, Sarasota, FL, USA) was used to fill the craniotomy and allowed to cure over several minutes. Cold-cure dental cement (A-M Systems Inc, Sequim, WA, USA) was applied to the skull to form a headcap, partially encapsulating the MEA site, bone screws, and extracranial ground and reference wires. The incision site was closed with surgical staples. After surgery, animals were injected with 0.05 mL/kg of cefazolin (Covetrus Inc., Portland, ME, USA) and 0.15 mL/kg of sustained-release buprenorphine (Zoopharm, Wedgewood Pharmacy Inc., Laramie, WY, USA). A follow-up buprenorphine injection with the same parameters was administered 72 h after surgery. The animals were allowed to recover for 7 days following surgery before the start of recordings.

### 2.3. Recording

Data were collected from animals weekly starting approximately one week following surgery and continued for 16 weeks post-surgery. Animals were anesthetized during recording using 2.5% isoflurane supplemented with pure oxygen. After anesthesia depth was confirmed using a toe pinch, the isoflurane level was reduced to 1.5–2.0% and data were then collected using an Omniplex recording system (Plexon Inc., Dallas, TX, USA). Wideband recordings of spontaneous activity in the motor cortex were collected for ten minutes from all 16 electrode channels simultaneously. In a subset of animals (*n* = 4), we examined the LFP power spectral density (PSD) for awake, freely behaving animals for both MEA types to verify the presence of all power bands 1- and 16-weeks post-implantation.

### 2.4. Electrochemical Assessment

In this study, the failure mode of interest was the foreign body response. However, it is well known that individual electrode sites on MEAs can experience disconnects after implantation due to mechanical or material failures [44,45]. For example, electrode metals may disconnect over time, interfering with the traces connecting the electrode site to the device back-end connector. Because of this, we used an electrochemical assay to monitor and exclude electrode sites showing signs of disconnections. A three-electrode cyclic voltammetry (CV) was performed as previously described [46] at slow (50 mV/s) and fast (50,000 mV/s) sweep rates. The cathodal charge storage capacities (CSCc) for each sweep rate were calculated as the time integral of the cathodic current in one complete CV cycle. Electrode channels that exhibited a low CSCc, typically less than 0.1 mC/cm^2^, and a CV shape characteristic of an open circuit were presumed to have broken connector wires and excluded from the study. Electrode channels were otherwise considered functional to the time point at which the two conditions were met but were excluded from analysis thereafter. In cases where an electrode channel appeared to be disconnected one week, but connected in subsequent weeks, the electrode channel was excluded only during the week the disconnection was observed.

### 2.5. Data Processing

The continuous wideband data from the electrophysiological recordings were analyzed using the chronux MATLAB toolbox as well as custom MATLAB (MATLAB R2021a, Mathworks, Natick, MA, USA) scripts. Continuous data were down-sampled to 2000 Hz by re-sampling the continuous signal at every 20^th^ time stamp, and then processed using a 4-pole, Butterworth bandpass filter with a passband from 1 to 500 Hz. The data were also processed using a notch filter at 60 Hz to remove line-frequency electrical noise [47].

The PSD was calculated in MATLAB in ten-second intervals using a multitaper method from the chronux MATLAB toolbox (mtspectrumc). This method estimates spectral content using non-arbitrary, orthogonal Slepian windowing functions to reduce variance and bias [47,48]. The resulting spectral content was then averaged across all intervals. The PSD for the total LFP band (1–500 Hz) and individual frequency bands were calculated, as defined in Table 1 [47]. For taper parameters, the time-half bandwidth product, which describes the separation between frequency components required to distinguish them, was set to 4 and the number of tapers was 7. The signal was zero-padded to the next highest power of 2 before performing the fast-Fourier transform. Zero-padding is the process of adding zeroes to the end of a signal so that the number of samples is equal to a power of two, allowing more accurate calculation of amplitudes of signal components.

### 2.6. Statistical Methods

Statistical analysis was performed in RStudio (Version 4.2.2; Boston, MA, USA). All values are expressed as mean ± standard error of the mean. The bandpower was averaged across all electrode channels within a single MEA. Data were analyzed for normality using the Shapiro–Wilk test and visually assessed using quantile-quantile plots. The mean total LFP band power recorded at each time point was compared between conventional and a-SiC devices using a two-way ANOVA followed by a post-hoc Tukey-test (HSD), correcting for multiple comparisons. Then, for each frequency band (e.g., delta, theta, etc.) a one-tailed Wilcoxon rank-sum test as a non-parametric alternative to a one-tailed *t*-test was conducted to test statistical significance at each discrete timepoint (1-, 8-, and 16-weeks post-implantation). A p-value of less than 0.05 was considered statistically significant. Figures were generated using Neuroexplorer (Nex Technologies, Colorado Springs, CO, USA), OriginLab (Northampton, MA, USA) and MATLAB. Outliers during each timepoint, defined as points distant from the nearest quartile by more than 1.5 times the interquartile range, were excluded from ANOVA analysis.

### 2.7. Immunohistochemistry

At the end of the 16 weeks of implantation, a pilot a-SiC rat was euthanized using intraperitoneal sodium pentobarbital injection (Virbac Corporation in Westlake, Westlake, TX, USA). Transcranial perfusion was carried out with 350 mL of 1X PBS, followed by 300 mL of 4% paraformaldehyde (PFA) (Sigma-Aldrich, Saint Louis, MO, USA). The MEAs were carefully removed, and the brain was extracted and stored in 4% PFA for 48 h. After the fixation, the tissue was embedded in a 4% agarose gel (Sigma-Aldrich, Saint Louis, MO, USA), and glued to the slicing platform of a vibratome (VT 1000S, Leica vibratome, Wetzlar, Germany) to obtain a 100 μm thick section. The slices were stored in PBS containing 0.01% (*w/v*) sodium azide (Sigma-Aldrich, St. Louis, MO, USA) at 4 °C overnight. The following day, the slices were treated with sodium borohydride (Sigma-Aldrich, St. Louis, MO, USA) for 30 min and washed with 1x PBS for an hour. Then, the samples were blocked and permeabilized using the blocking buffer containing 4% normal goat serum (Abcam Inc., Cambridge, UK), 0.3% Triton X-100 in 1X PBS with 0.01% sodium azide (Sigma-Aldrich, St. Louise, MO, USA) for an hour. The slices were then washed 3 times in 1x PBS with sodium azide. Three drops of Image-iT were added to the wells and washed after 30 min with 1x PBS and sodium azide. The samples were then treated with primary antibodies reconstituted in the blocking buffer and stored at 4 °C overnight. The following primary antibodies were used: NeuN (Abcam Inc., 1:500) and GFAP (Abcam Inc., 1:500). The next day, samples were washed with 1x PBS, then with 1X PBS containing 0.2% Triton X-100, and another wash with 1X PBS each for 15 min. Afterward, samples were treated with secondary antibodies for 2 h. The following secondary antibodies were used: Goat anti-mouse Alexa Fluor 555 (Abcam Inc., 1:4000) and Goat anti-chicken Alexa Fluor 647 (Abcam Inc., 1:4000). Again, the samples were cycled through the 15-min washes of 1X PBS, 1X PBS + 0.2% Triton X-100, and 1X PBS. Next, the slices were mounted on glass slides using Fluoromount aqueous mounting medium (Sigma-Aldrich, St. Louis, MO, USA) and stored at 4 °C until imaging. Images were taken using confocal microscopy (Eclipse Ti, Nikon, Tokyo, Japan) at 10× magnification.

## 3. Results

### 3.1. LFP Recording and Spectrogram

**Figure 1:**
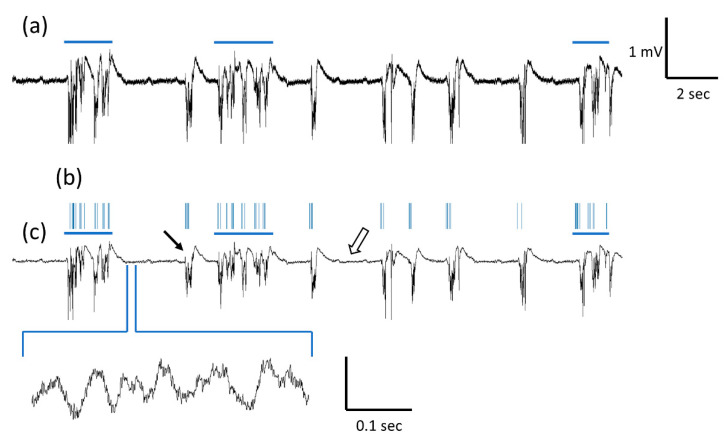
Representative continuous electrophysiological recordings from a-SiC MEAs one-week post-implantation. (**a**) Raw signal before filters are applied. Blue horizontal line denotes simultaneous single unit activity. (**b**) Raster plot showing the time of single unit activity. (**c**) Low-pass (1–500 Hz) filtered data showing the total LFP band. Black arrows point to intermittent multiphasic waveforms. Inset shows a zoomed-in version of representative low amplitude oscillations (white arrow).

Figure 1 shows a representative a-SiC MEA continuous recording one-week post-implantation. Figure 1a shows raw continuous signal. Figure 1b shows continuous signal after applying the 500 Hz low pass-filter to observe the LFP band. Traces show intermittent 1–2 mV amplitude, multiphasic waveforms lasting less than 1 s (black arrow). As highlighted by comparing Figure 1b,c, multiphasic waveforms were largely coincident with multi-unit bursting (blue horizontal line), which shows the low-pass and band-pass filtered data aligned. In addition, recordings showed lower amplitude oscillations of approximately 20–50 µV peak-to-peak (white arrow).

### 3.2. Power Spectral Density

**Figure 2:**
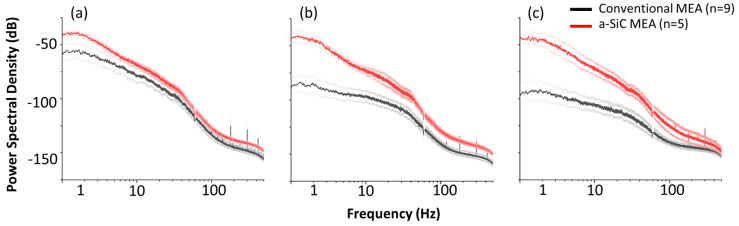
Comparative power spectral densities (PSD) for a-SiC MEAs and conventional planar, silicon-based devices. PSD (**a**) 1 week, (**b**) 7 weeks, and (**c**) 16 weeks post-implantation for conventional and a-SiC MEAs. Bold red (a-SiC) and grey (conventional) traces represent the mean and light traces represent the standard error of the mean.

Figure 2 shows the PSD of recordings from conventional and a-SiC MEAs, averaged across all functional electrode channels from all animals, on weeks 1, 7, and 16 post-implantation. Two electrode sites on the conventional MEAs out of 144, and one a-SiC electrode site out of 80 were excluded (two electrode sites from week 1 post-implantation to the end of the study, one electrode site from week 4 post-implantation to the end of the study) based on electrochemical analysis described above, as device failure resulting from connection issues was not the failure mode of interest in this study. This resulted in the exclusion of <2% of electrode sites. Both MEA types showed lower power in the alpha, beta, and gamma bands (>10 Hz) compared to delta and theta bands. This may be partially due to the light anesthesia used during recording, as the activity of the higher frequency bands is related to functions such as conscious perception and information processing, which do not occur during anesthesia [49]. Data at 60 Hz were removed by a notch filter as described above and corresponding datapoints were excluded from Figure 2. We observed that 1-week post-implantation, conventional and a-SiC MEAs exhibited comparable spectral content around the delta, theta, and alpha bands with the maximum PSD showing in the delta band at approximately −50 dB. Eight weeks post-implantation, we observed a decline in maximum PSD in the conventional MEAs. The maximum PSD in the delta band was approximately −80 dB. There was no corresponding decline in PSD in a-SiC MEAs, and the maximum PSD remained at approximately −50 dB. Finally, 16 weeks post-implantation we observed a shift of the most prominent frequency band from delta to theta for a-SiC devices; however, the PSD was comparable to 1-week post-implantation (−50 dB). In contrast, conventional devices experienced a marked loss of PSD on all frequency bands (<100 dB), suggesting failure of the neural interface. Finally, electrochemical characterization prior to implantation showed not statistically significant differences (*p* = 0.12) in 1 kHz impedance between a-SiC (0.58 ± 0.20 MΩ) and conventional devices (0.29 ± 0.03 MΩ).

### 3.3. Bandpower

**Figure 3:**
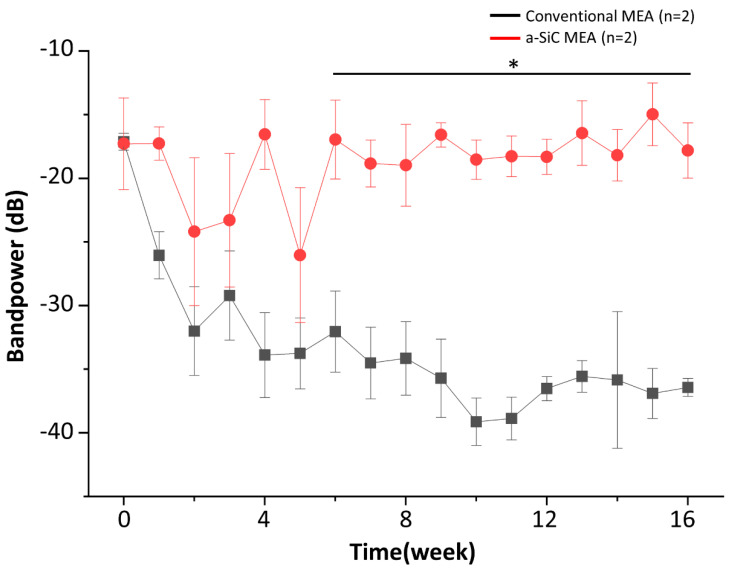
Total LFP bandpower recorded from a-SiC and conventional MEAs for up to 16-weeks post-implantation. The span of the horizontal bar shows that statistical significance between a-SiC and conventional MEAs was achieved at all timepoints under the bar, from week 6 post-implantation to week 16. Data reported as mean ± SEM; statistical significance: * *p* < 0.05.

Figure 3 shows the average bandpower for the total LFP frequency band each week up to 16 weeks post-implantation. Both MEA types are comparable at the time of implantation (−19.9 ± 2.86 dB for conventional vs. −20.7 ± 3.53 dB for a-SiC MEAs; *p* = 0.92). Conventional devices experience a rapid decline in PSD as early as 2-weeks post-implantation. By week 8 post-implantation, the power loss experienced by conventional MEAs had progressed by close to −10 dB (−35.71 ± 3.080 dB), while the power remained constant for a-SiC devices (−16.59 ± 0.9593 dB). The power for conventional devices continued to decline to week 16 post-implantation (−46.73 ± 3.48 dB), whereas the a-SiC MEAs maintained a constant power (−17.83 ± 2.17 dB). These differences observed with sustained statistical significance (*p* ≤ 0.05) at week 6 post-implantation, supporting the use of a-SiC devices for reliable chronic LFP recordings.

The ANOVA analysis and Tukey HSD post-hoc test indicated a statistically significant effect of MEA type on LFP bandpower (*p* < 0.0001). Twelve data points (approximately 6%) in the total LFP band were identified as outliers and excluded from analysis. All data were normally distributed after outliers were removed.

Table 2 shows the bandpower mean ± SEM obtained with both types of MEAs at weeks 0 (time of implantation), 8, and 16 post-implantation. Statistical significance in all reported bands emerged by week 8 post-implantation.

### 3.4. Awake LFP Recordings

**Figure 4:**
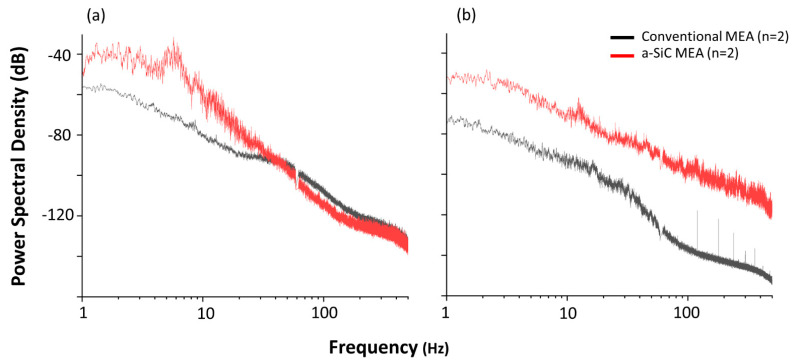
Comparative power spectral densities (PSD) for a-SiC MEAs and conventional devices. In awake, freely moving rats. Average PSD for a-SiC (*n* = 2) and conventional MEAs (*n* = 2) at (**a**) 1 weeks (**b**) and 16 weeks post-implantation.

Figure 4 shows the PSD from a subset of animals recorded while awake and freely exploring a recording cage. At the beginning of the study in week 1, we observed an increase of the alpha (peak at −30 dB) frequency power band in the a-SiC device, which was not present in the anesthetized recordings. For the conventional devices, the alpha band was also more prominent (−80 dB) compared to anesthetized recordings but was approximately 30 dB lower than a-SiC devices. The a-SiC MEAs also demonstrated an increase in the beta (peak at −50 dB) frequency band, which was not present in the anesthetized recordings. In contrast, there was no apparent increase in conventional devices for the beta band compared to anesthetized recordings. After 8 weeks post implantation, the alpha band for both types of devices became less prominent (a-SiC: −50 dB and conventional: −80 dB), while the beta band in a-SiC devices remained largely consistent (−50 dB). These findings are consistent with the overall loss of total LFP bandpower [50].

### 3.5. Immunohistochemistry

**Figure 5:**
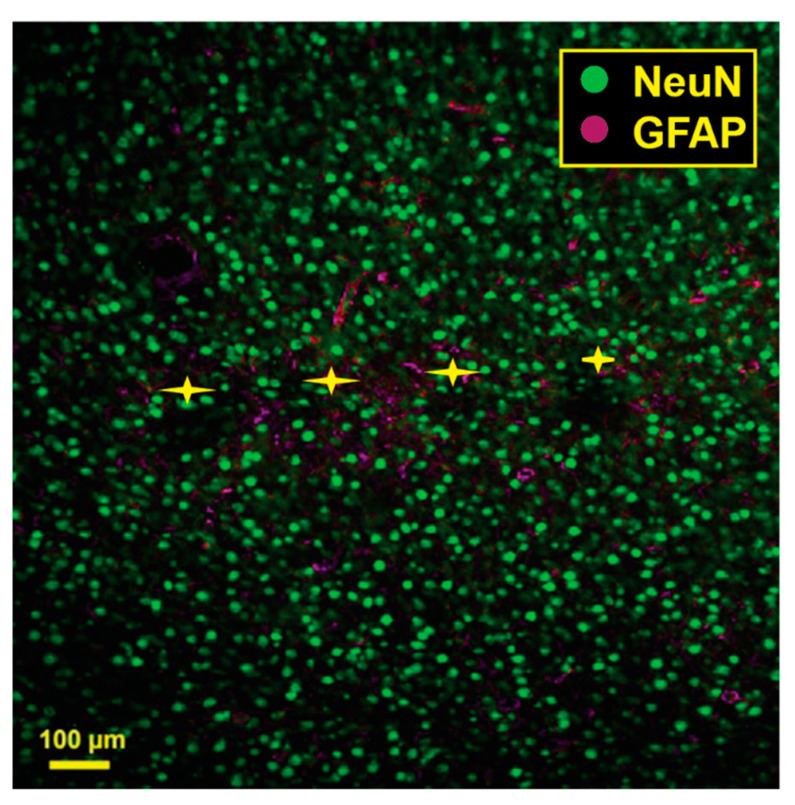
Pilot histological analysis a-SiC MEA (*n* = 1). Green represents neuronal nuclear antigen (NeuN) and magenta represents glial fibrillary acidic protein (GFAP). The yellow stars represent the putative implant location for each one of the four shanks.

Figure 5 shows the results of a pilot histological analysis of a brain implanted with an a-SiC MEA. A comprehensive analysis of histological response to a-SiC and conventional MEAs is ongoing.

## 4. Discussion

In this study, we report the PSD of LFPs in the rat motor cortex from ultra-thin a-SiC MEAs compared to silicon-based conventional MEAs. Previous research has explored the design and fabrication of ultra-thin MEAs from materials such as carbon fibers [51,52,53] to reduce foreign body response, with the goal of creating a device capable of stable, long-term neural recordings. These reduced cross-sectional area devices have also shown improvements in the severity of glial scarring and neuronal density compared to conventional devices [50]. While the improved performance of a-SiC MEAs compared to conventional MEAs observed in this study can be attributed, at least in part to the reduced cross-sectional area, other factors likely contribute to the improvement. Ultra-thin a-SiC MEAs are highly flexible due to the small cross-sectional dimensions [41]. An increased degree of flexibility has been shown to promote neuron survival in the vicinity of chronically implanted MEAs [54].

For the conventional MEAs, we observed a sharp decline in total LFP bandpower in the first two weeks of the study (8 dB/week), followed by a slower decline (0–2 dB/week) and eventually reaching as much as 20 dB below the initial bandpower. This bandpower decline appears to correlate temporally with the neuronal loss and tissue encapsulation resulting from both acute injury during device implantation and the foreign body response, as reported elsewhere [6,7,8,27,55]. In contrast, a-SiC MEAs show stable total LFP bandpower over the chronic implantation period with −20.7 ± 3.53 dB at the beginning and −17.8 ± 2.17 dB at the end of the study. This may be explained, at least in part, by the small cross-sectional area of ultra-thin a-SiC MEAs, which can minimize the foreign body response, improving neuronal survivability [29]. Preservation of neuronal cells has a direct effect on the total LFP bandpower because neuronal synaptic activity contributes to the extracellular fields that are recorded within this band [19]; if the synaptic activity decreases due to neuronal loss, the total LFP bandpower also decreases. These findings are consistent with previous literature showing that electrodes with small cross-sectional areas are more capable of long-term stable neural recordings than current silicon-based, planar conventional devices [51]. However, there is an apparent decrease in the total LFP bandpower between weeks 2 and 5 for a-SiC MEAs, which coincides with temporal patterns of oligodendrocyte degeneration, neuronal loss, and wound healing [56,57,58]. This bandpower decrease returns to baseline levels 6 weeks post-implantation. This could be an indication that the neuroinflammation process has largely been resolved but needs to be confirmed in future studies.

We noted that the PSD observed here during anesthetized recordings was most prominent in frequency bands below 10 Hz, corresponding to delta, theta, and low alpha frequency bands. This may be caused by the use of light anesthesia during recordings resulting in the complete suppression of the high alpha, beta, low gamma, and high gamma bands (≥10 Hz). These observations are consistent with findings in the literature because activity of LFP bands above 10 Hz is associated with conscious processes that do not occur during anesthesia [19,49,59,60]. Because of this, we performed recordings in a small subset (*n* = 2 for each group) of awake, freely behaving animals to confirm the feasibility of recording neural activity associated with these conscious processes at points 1- and 16-weeks post-implantation. During the awake recordings, the animals were freely exploring in a small cage. The presence of the alpha and beta bands became apparent in the a-SiC MEA but there was no prominence of the beta band in conventional MEAs, even 1-week post-implantation. These findings suggest the ability of a-SiC ultra-thin devices to record bands associated with motor planning, further supporting their use for brain-machine interface applications. However, a limitation of this awake study was the small sample size included. This will be addressed in future studies that explore the development of chronic brain-machine interfaces, where a sufficiently powered study will investigate the chronic stability of behaving neural signals using a-SiC devices. Furthermore, a complete histological analysis of the effects of implantation with a-SiC MEAs, as well as single unit analysis are undergoing. Previous work has shown that there is an inverse relationship between LFP power and foreign body response, where glial encapsulation can reduce the LFP power [38]. We expect that the undergoing study will demonstrate that a-SiC devices have a similar or reduced foreign body response compared to conventional devices, which would explain the reduction of LFP power.

The relatively low channel count (16 electrode sites) of the devices used in this study is a limitation for their use in brain-machine interfaces, which benefit from high-channel counts for neuronal decoding [25]. Our group is currently investigating strategies to increase channel density, while maintaining a small cross-sectional dimension, which may include electron beam lithography [61,62] and multilayer metallization [63,64].

In this study, we used multi-shank arrays that have the ability to record from multiple cortical layers and columns simultaneously, which has been suggested as beneficial for achieving high decoding accuracy [21]. However, the quality of neural recordings acquired from multi-shank devices have been previously shown by our group [65] to decline faster than single-shank devices, potentially due to additional mechanical trauma induced during the simultaneous implantation of spatially parallel shanks [41]. The use of ultra-thin a-SiC devices appears to preserve neural signal recordings in the total LFP band beyond 6 weeks, compared to conventional devices. This further supports the use of the a-SiC devices for chronic decoding in brain-machine interfaces.

## 5. Conclusions

Overall, we have demonstrated the feasibility of ultra-thin a-SiC devices to reliably record LFPs for up to 16 weeks post-implantation in the rat cortex compared to conventional devices. These results support the use of a-SiC MEAs for chronic brain-machine interfaces, where they may provide improved chronic decoding accuracy due to signal quality preservation. However, future work, will focus on analyzing the power of LFPs during a behavioral task to determine whether signals relevant to decoding are also preserved. Furthermore, standalone single unit analysis, in combination with LFPs will be used to determine chronic decoding accuracies with a-SiC MEAs.

## Figures and Tables

**Table 1:** LFP bandwidths for calculation of PSD.

Band	Frequencies (Hz)
Total LFP band	1–500
Delta	1–4
Theta	4–8
Alpha	8–13
Beta	13–31
Low gamma	31–59
High gamma	61–100

**Table 2:** Bandpower for weeks 1, 7, and 16 post-implantation and statistical comparisons between groups. Data reported as mean ± SEM; statistical significance: * *p* < 0.05.

Band	Week 0			Week 7			Week 16		
	a-SiC	Conventional	*p*	a-SiC	Conventional	*p*	a-SiC	Conventional	*p*
Total LFP (dB)	−20.7 ± 3.53	−19.9 ± 2.86	ns	−21.1 ± 3.2	−34.1 ± 2.89	*	−17.8 ± 2.17	−36.4 ± 0.71	*
Delta (dB)	−22.1 ± 3.53	−22.3 ± 3.82	ns	−19 ± 2.53	−40.7 ± 3.84	*	−19.9 ± 2.43	−43.6 ± 1.58	*
Theta	−29.2 ± 4.08	−28.2 ± 3.08	ns	−27.7 ± 4.6	−43.3 ± 3.84	*	−25.5 ± 2.15	−45.6 ± 1.09	*
Alpha (dB)	−33.5 ± 4.07	−32.3 ± 3.21	ns	−30.6 ± 5.13	−44.2 ± 3.3	*	−30.3 ± 1.17	−46.7 ± 1.22	*
Beta (dB)	−34.0 ± 3.33	−34.9 ± 2.35	ns	−31.8 ± 2.45	−42.4 ± 3.12	*	−30.3 ± 1.18	−45.2 ± 1.4	*
Low gamma (dB)	−42.2 ± 3.16	−38.8 ± 2.11	ns	−37.6 ± 2.17	−46.3 ± 2.59	*	−36.8 ± 0.7	−50.3 ± 1.09	*
High gamma (dB)	−50.7 ± 2.67	−45.6 ± 1.06	ns	−45.7 ± 1.41	−51.9 ± 1.65	*	−44.7 ± 1.25	−54.3 ± 0.76	*

## Data Availability

Data available upon request.
